# Extreme Anticlockwise Endoscopist Repositioning for Biliary Cannulation in Left-wall Periampullary Diverticulum

**DOI:** 10.1055/a-2916-0033

**Published:** 2026-08-12

**Authors:** Wai Liam Lam, Nasir Javed, Muhammed Saad, Shabbir Jivanjee, Kar Lau, Roderick Prawiradiradja, Damien Durkin, Srisha Hebbar

**Affiliations:** 1 Department of Gastroenterology and Endoscopy 9898 University Hospitals of North Midlands NHS TrustStoke-on-TrentEnglandUnited Kingdom of Great Britain and Northern Ireland

**Keywords:** pancreatobiliary (ERCP/PTCD), ERC topics, quality and logistical aspects, training, performance and complications

## Abstract

**Background and Study Aims** A papilla located on the left wall of a periampullary diverticulum creates a mechanically unfavourable axis for biliary cannulation that is not readily corrected from the conventional endoscopist position. We describe the Extreme Anticlockwise Bileduct Cannulation Diverticulum (ABCD) technique, where “Extreme” qualifies the degree of anticlockwise rotation applied—an endoscopist-repositioning manoeuvre designed to address this constraint without additional instrumentation.

**Patients and Methods** Following observations in two index cases, the technique was adopted pre-emptively in consecutive patients with a confirmed left-wall periampullary papilla. Twenty-four patients were prospectively enrolled between July 2021 and February 2026. The endoscopist repositioned to the patient’s head end and applied extreme anticlockwise duodenoscope rotation to realign the instrument axis. The primary outcome was successful biliary cannulation. Adverse events were graded using the Cotton consensus criteria.

**Results** Successful biliary cannulation was achieved in 23/24 patients (95.8%), all within 5 min of adopting the repositioned stance. Primary guidewire cannulation was achieved in 20/24 (83.3%); the remaining three required needle-knife fistulotomy, performed from the same repositioned stance. Trainees were involved in 22 cases and cannulated independently in 16 (72.7%). Then, 3 of 24 patients (12.5%) experienced post-sphincterotomy bleeding, graded as AGREE Grade I. No post-endoscopic retrograde cholangiopancreatography (ERCP) pancreatitis or other major complications were observed.

**Conclusions** The Extreme ABCD technique is a simple, equipment-independent repositioning strategy for ERCP in left-wall periampullary diverticular anatomy. In this prospective series, it was associated with a high rate of biliary cannulation without rescue accessories and was feasible in a supervised training environment. Multicentre evaluation is warranted.

## Introduction

Endoscopic retrograde cholangiopancreatography (ERCP) remains a key therapeutic modality for biliary and pancreatic disease, and selective biliary cannulation is its defining technical step. Periampullary diverticula are encountered in 10–30% of ERCP procedures and represent a common cause of difficult cannulation. 1 The impact of diverticula depends critically on papillary position, with left-wall papillae presenting a distinct mechanical challenge.

In this configuration, the orientation of the duodenoscope elevator and tip deflection opposes the desired cannulation axis when approached from the conventional endoscopist position. Published instrument-assisted cannulation strategies, including traction devices, dual-instrument techniques, and specialised forceps, 2 3 4 5 6 7 each of which addresses the challenge through additional equipment, increasing procedural complexity and resource utilisation. None directly modifies the operator-instrument relationship that underlies the mechanical constraint.

We describe the Extreme Anticlockwise Bileduct Cannulation Diverticulum (ABCD) technique, an endoscopist-repositioning manoeuvre that addresses this relationship. The technique arose from initial observation in two index cases, after which it was adopted pre-emptively in consecutive patients with this anatomy. We report outcomes from a prospective series and evaluate feasibility in both experienced and trainee endoscopists, illustrated by two procedural videos.

## Patients and Methods

### Study Design and Setting

A prospective observational case series was conducted at a single tertiary referral centre (University Hospital of North Midlands NHS Trust, Stoke-on-Trent, UK) between July 2021 and February 2026. The study was conducted as a service evaluation in accordance with institutional policy. Written informed patient consent was obtained for publication of procedural video and images in all cases.

### Index Discovery

The technique was not planned but arose from observation during two consecutive cases performed within the same week, in both of which standard cannulation of a left-wall papilla had exceeded 5 min. In both cases, the lead endoscopist had inadvertently repositioned to the patient’s head end and rotated away from the primary monitor, facing the secondary screen behind them, arriving in the same stance on each occasion. Biliary cannulation was achieved in both cases. Recognising the reproducibility of this positional advantage, the technique was adopted pre-emptively for all subsequent cases with this anatomy. These two index cases are not included in the outcome series.

### Patient Selection

Consecutive patients undergoing ERCP in whom a papilla was identified on the left wall of a periampullary diverticulum at initial duodenoscopy were included. Patients with surgically altered upper gastrointestinal anatomy were excluded. Papillary position was determined intra-procedurally by the lead endoscopist (S.H.). The technique was applied pre-emptively from the outset of cannulation once left-wall anatomy was confirmed; all cases met the European Society of Gastrointestinal Endoscopy (ESGE) definition of difficult cannulation. 8 No formal classification system was applied during the study period; using the Boix classification, 9 10 the cases in this series would correspond to Type Ib (papilla on the left wall inside the diverticulum), Type IIa (papilla at the apical left margin), or Type IIc (papilla at the centre left margin), depending on the precise position of the orifice relative to the diverticular rim. All were grouped together under the operational descriptor of “left-wall periampullary papilla” on the basis that each configuration imposes the same unfavourable leftward cannulation axis that the Extreme ABCD technique is designed to correct. Representative endoscopic appearances of the left-wall papilla, both before and after repositioning, are demonstrated in the accompanying video. This grouping represents a methodological limitation, and prospective multicentre evaluation will apply formal Boix classification to enable more granular anatomical characterisation and facilitate cross-study comparisons.

### The Extreme ABCD Technique



**Video 1** Extreme ABCD technique performed by the lead endoscopist and by a supervised trainee endoscopist (>100 independent ERCPs): left-wall papilla identified; endoscopist repositioned to head end facing secondary monitor; extreme anticlockwise rotation applied; en-face papillary realignment achieved; biliary cannulation confirmed fluoroscopically within 5 min.


All procedures were performed with the patient in the standard left semi-prone position under conscious sedation with intravenous midazolam and fentanyl, administered and monitored by a trained endoscopy nurse, endoscopy trainee, or the lead endoscopist depending on case circumstances. Fluoroscopy was controlled by a radiographer. Repositioning of the endoscopist to the head end of the table required no change to the patient’s position or sedation monitoring arrangement; the nurse remained at the patient’s side throughout, and the radiographer operated the fluoroscopy pedal from a fixed position unaffected by the endoscopist’s stance.

The technique is demonstrated in Video 1 (lead endoscopist and supervised trainee). After identifying the left-wall papillary position, the endoscopist repositioned from the conventional left-sided stance to stand at the patient’s head end, then rotated their own body away from the primary monitor to face the secondary screen positioned behind them. In dual-monitor ERCP suites, this screen is located at the head end of the table; in units with a single monitor, repositioning the primary monitor further to the patient’s left minimises ergonomic compromise during body rotation. This stance enables extreme anticlockwise torque to be applied naturally to the duodenoscope shaft, translating the tip laterally within the second part of the duodenum and realigning the elevator and instrument channel with the left-wall papilla. Selective biliary cannulation was then performed using a standard sphincterotome and a hydrophilic guidewire. In three cases where primary guidewire cannulation was not achievable, needle-knife fistulotomy was performed from the same repositioned stance without reverting to the standard positioning. No rescue accessories were used in any case.

### Trainee Involvement

Then, 22 of the 24 cases were performed by trainee endoscopists who had each independently performed more than 100 ERCPs prior to participation. In all 22 cases, the lead endoscopist (S.H.) was scrubbed and actively guiding throughout the procedure. The positional principle was explained and the repositioning demonstrated before cannulation commenced.

### Outcome Measures

The primary outcome was successful biliary cannulation, defined as guidewire and catheter passage into the common bile duct confirmed fluoroscopically. Time to cannulation was recorded prospectively from the adoption of the repositioned stance. All patients received rectal diclofenac 100 mg for post-ERCP pancreatitis prophylaxis in accordance with local institutional protocol. Adverse events were graded using both the Cotton–Eisen lexicon 11 and the AGREE (Adverse events in GI Endoscopy) classification. 12 Post-ERCP pancreatitis (PEP) was defined using the revised Atlanta criteria for acute pancreatitis, requiring two of the following: characteristic abdominal pain lasting >24 h, serum amylase or lipase ≥3 times the upper limit of normal, and characteristic findings on cross-sectional imaging. All patients were followed up at 30 days via hospital records and direct patient contact.

## Results

Twenty-four consecutive patients with a left-wall periampullary papilla were enrolled between July 2021 and February 2026. The mean age was 76.4 years (SD ± 8.1 years; 15 were male). The primary indication was choledocholithiasis in 21 patients (87.5%), bile leak in two patients (8.3%) and biliary stricture in one patient (4.2%). Full demographic, procedural, and outcome data are presented in Table 1 .

**Table 1:** Patient demographics, procedural details, and outcomes ( *n* = 24 prospective cases).

*Demographics*	
** Patients, *n***	24
**Age, mean (SD), years**	76.4 (±8.1)
** Male sex, *n* (%) **	15 (62.5%)
***Indications***	
** Choledocholithiasis, *n* (%) **	21 (87.5%)
** Bile leak, *n* (%) **	2 (8.3%)
** Biliary stricture, *n* (%) **	1 (4.2%)
***Procedural details***	
**Study period**	July 2021–February 2026
** Technique applied pre-emptively, *n* (%) **	24/24 (100%)
** Cases with trainee involvement, *n* (%) **	22/24 (91.7%)
** Trainee independent cannulation, *n* (%) **	16/22 (72.7%)
** Inadvertent PD cannulation, *n* (%) **	1 (4.2%)
** Rescue accessories used, *n* (%) **	0 (0%)
***Outcomes***	
** Cannulation success, *n* (%) **	23/24 (95.8%)
** Cannulation within 5 min, *n* (%) **	23/24 (95.8%)
** Standard guidewire cannulation, *n* (%) **	20/24 (83.3%)
**Needle-knife fistulotomy**	3/24 (12.5%)
** Post-ERCP pancreatitis, *n* (%) **	0 (0%)
** Mild post-sphincterotomy bleeding, *n* (%) **	3 (12.5%)
**Major complications**	None
**Unplanned hospital admission**	None
**30-day mortality**	None

The Extreme ABCD technique was adopted pre-emptively from the outset in all 24 patients. Successful biliary cannulation was achieved in 23/24 patients (95.8%), all within 5 min of adopting the repositioned stance (measured from the point of repositioning, not from the start of the procedure). The single failure occurred in the setting of an intradiverticular papilla where the orifice could not be visualised despite repositioning; this patient subsequently underwent endoscopic ultrasound-guided biliary drainage.

Twenty-two procedures were performed by trainee endoscopists under direct scrubbed supervision, achieving successful biliary cannulation in 16/22 cases (72.7%). In the remaining six cases, the lead endoscopist took over cannulation and achieved biliary access successfully, contributing to the overall 95.8% procedural cannulation rate. Inadvertent pancreatic duct (PD) cannulation occurred in one patient (4.2%) during initial guidewire manipulation; the wire was withdrawn and biliary cannulation achieved on the next attempt without resort to double-wire technique. No rescue accessories were required in any patient.

Then, 3 of the 24 patients (12.5%) experienced post-sphincterotomy bleeding, all managed endoscopically at the time of the procedure without clinical sequelae, haemoglobin drop, or need for transfusion. Under the AGREE classification, 12 all three were Grade I (adverse event that resolved without any additional intervention beyond the index endoscopic procedure). No post-ERCP pancreatitis meeting the revised Atlanta criteria was observed. All patients were followed up at 30 days; none developed characteristic PEP symptoms, and no patient had imaging performed that demonstrated pancreatic inflammation. All patients received rectal diclofenac 100 mg for prophylaxis. There were no cases of perforation or cholangitis. No patient required unplanned hospital admission, and there was no 30-day procedure-related mortality.

## Discussion

In this prospective consecutive series, the Extreme ABCD technique was associated with a high rate of successful biliary cannulation in patients with left-wall periampullary diverticulum, without recourse to rescue accessories. This study was not designed to demonstrate superiority over conventional positioning, but to describe the feasibility and outcomes of a pre-emptive positional approach in this anatomical setting.

The technique addresses a key mechanical limitation of standard ERCP positioning. Repositioning the endoscopist to the patient’s head end and facing away from the primary monitor allows controlled application of extreme anticlockwise torque, resulting in lateral displacement of the duodenoscope tip and improved alignment between the instrument channel and the left-wall papilla. This contrasts with instrument-assisted strategies, which compensate for unfavourable geometry without modifying the underlying operator–instrument relationship. The recent SOCCER randomised controlled trial demonstrated that SpyBite forceps-assisted cannulation achieved a 100% success rate versus 83.9% without forceps in difficult cannulations in a broader population including but not limited to periampullary diverticulum 7 ; the Extreme ABCD technique compares favourably with published accessory-based approaches, although direct comparison is not possible. The mechanical basis of the repositioning manoeuvre is based on procedural observation and has not been formally quantified.

The pre-emptive use of this manoeuvre is a distinguishing feature. By altering positioning at the outset once left-wall anatomy is recognised, repeated attempts in a mechanically unfavourable orientation may be avoided, potentially reducing pancreatic trauma and the risk of complications from repeated blind sphincterotome manipulation adjacent to the diverticulum. It is acknowledged that some patients, particularly those with a left-wall papilla at the diverticular edge rather than entirely within the diverticulum, might have been successfully cannulated from the standard position; this cannot be excluded in a pre-emptive single-arm design.

Twenty-two procedures were performed by trainee endoscopists who had each independently completed more than 100 ERCPs, under direct scrubbed supervision. In sixteen cases (72.7%), trainees achieved successful cannulation independently; in the remaining six, the lead endoscopist assumed cannulation and succeeded in all, with the overall procedural success rate remaining 95.8%. This suggests feasibility in a supervised training environment and indicates the technique can be conveyed and adopted readily once the positional principle is understood. However, independent performance and generalisability to less experienced trainees cannot be inferred from this series.

The zero rate of post-ERCP pancreatitis is reassuring, particularly given the inadvertent PD cannulation in 4.2% of patients and the absence of prophylactic PD stenting in any case. All patients received rectal diclofenac as per protocol; its contribution to the zero PEP rate cannot be excluded. The bleeding rate of 12.5% warrants contextualisation. All three episodes lacked clinical sequelae (no haemoglobin drop, no transfusion, no unplanned admission) and were graded as Grade I under the AGREE classification 12 —the lowest severity tier, requiring no additional intervention beyond the index procedure. Under the Cotton–Eisen lexicon, which requires clinical evidence of bleeding beyond endoscopic appearances, haemoglobin drop of 3 g/dL or more, and need for transfusion to qualify as mild bleeding, none of these episodes met the threshold; the endoscopic haemostasis rate was therefore 0% by that definition. The rate of 12.5% reported in the table reflects the operational AGREE Grade I classification and is consistent with published rates for therapeutic ERCP with sphincterotomy and balloon trawl in an elderly cohort.

Limitations include the single-centre design, absence of a contemporaneous comparator group, and the operational grouping of Boix Types Ib, IIa, and IIc under a single “left-wall” descriptor rather than formal prospective classification—as detailed in the Methods. All procedures were performed or supervised by a single experienced endoscopist, which may limit generalisability. Applicability in the prone ERCP position and across different ERCP suite configurations requires further evaluation. Multicentre prospective validation is planned.
